# Effect of continuous positive airway pressure on carotid intima-media thickness in patients with obstructive sleep apnea: A meta-analysis

**DOI:** 10.1371/journal.pone.0184293

**Published:** 2017-09-01

**Authors:** Li-Da Chen, Li Lin, Xue-Jun Lin, Yang-Wu Ou, Zhi Wu, Yu-Ming Ye, Qiao-Zhen Xu, Ya-Ping Huang, Zhi-Ming Cai

**Affiliations:** 1 From Department of Respiratory Medicine, Zhangzhou Affiliated Hospital of Fujian Medical University, Xiangcheng District, Zhangzhou, Fujian Province, People's Republic of China; 2 From Department of Laboratory Medicine, Zhangzhou Affiliated Hospital of Fujian Medical University, Xiangcheng District, Zhangzhou, Fujian Province, People's Republic of China; University of Rome Tor Vergata, ITALY

## Abstract

**Objective:**

Obstructive sleep apnea (OSA) is associated with increased carotid intima-media thickness (IMT), an early marker of atherosclerosis. Continuous positive airway pressure (CPAP) is the first-line treatment for OSA. A meta-analysis was performed to determine whether CPAP therapy could decrease carotid IMT.

**Methods:**

The PubMed, Embase, Web of Science, and Cochrane library were searched before March, 2017. Weighted mean difference (WMD) was calculated to estimate the treatment effects of pre and post-CPAP therapy. Seven studies were examined and the meta-analysis was performed using STATA 12.0.

**Results:**

There was no change of carotid IMT before and after CPAP treatment in OSA patients (WMD = 0.052, 95% confidence interval (CI) = −0.002 to 0.105, z = 1.90, p = 0.057). Meanwhile, meta-analysis of the two RCTs showed that carotid IMT was not changed in CPAP group when compared with control group (WMD = 0.002 95% CI = −0.125 to 0.129, z = 0.03, p = 0.976). Subgroup analyses indicated that carotid IMT was significantly decreased after CPAP use in more severe OSA patients (AHI≥50) (WMD = 0.073, 95% CI = 0.022 to 0.124, z = 2.80, p = 0.005) and patients with therapeutic duration ≥6 months (WMD = 0.121, 95% CI = 0.019 to 0.223, z = 2.32, p = 0.021).

**Conclusions:**

CPAP had no impact on carotid IMT in OSA patients. However, carotid IMT was significantly decreased after CPAP treatment in more severe OSA patients and patients with longer CPAP usage.

## Introduction

Obstructive sleep apnea (OSA) is the most common type of sleep disorder characterized by repetitive episodes of partial or complete upper airway obstruction during sleep [1]. It affects 4% of adult men and 2% of adult women [2]. OSA has been shown to be an important independent risk factor for cardiovascular diseases and cognitive impairment [3, 4]. Various different mechanisms are involved in the pathogenesis of cardiovascular disorders and cognitive impairment in OSA patients.

Carotid intima-media thickness (IMT) is defined as the distance between the lumen-intima interface and the media-adventitia interface of the artery wall. Carotid IMT is an early marker of atherosclerosis and predicts future cardiovascular events [5, 6]. Meanwhile, some investigations have suggested that the presence of a severe carotid stenosis or occlusion negatively influences cognitive performances [7, 8]. Many studies have investigated the correlation between OSA and carotid IMT measurements and have demonstrated that severity of OSA is independently related to the carotid IMT [9–11].

Continuous positive airway pressure (CPAP) is currently recognized as the first line therapy of OSA. Previous studies have shown conflicting results on the effect of CPAP on carotid IMT in patients with OSA [12–15]. Some studies demonstrated the favorable effect of CPAP on carotid IMT [12, 13], while others did not [14, 15]. Meta-analysis is a reliable way to address discrepancies between studies; therefore, we conducted a meta-analysis to evaluate the efficacy of CPAP on carotid IMT among patients with OSA.

## Materials and methods

### Search strategy

The Embase, PubMed, Cochrane Library and Web of Science were searched from inception to March 29th, 2017 for related literature. Searches combined free-text and MeSH terms, and the following search terms were used: “sleep apnea or sleep disordered breathing” and “continuous positive airway pressure or CPAP” and “carotid intima-media thickness”. The reference lists of the retrieved articles were checked to identify additional papers. Two researchers independently identified the eligible studies.

### Inclusion/Exclusion criteria of literature

We selected studies that met the following inclusion criteria: 1. The published article had to be in English; 2. All subjects were adults (>18 y) with newly diagnosed OSA; 3. CPAP was applied; 4. Measuring the carotid IMT by ultrasound; 5. Mean and SD (or SE) of common carotid IMT must be reported before and after CPAP therapy. All case reports, case series, abstracts, editorials, conference articles, letters, animal studies and review articles were excluded. If any of the required data could not be found in the published report, the corresponding author was contacted to provide the missing data of interest. Any disagreement between the two reviewers was resolved after additional discussion.

### Data extraction

The following information was abstracted and evaluated independently by two investigators, including the first author, publication year, country of the study, the number of patients, gender distribution, patient inclusion criteria, study design, mean daily CPAP usage time, duration of CPAP therapy, carotid IMT measurement, participant characteristics, common carotid IMT before and after CPAP treatment.

### Statistical analysis

The meta-analysis was conducted using Stata Version 12.0 for Windows (Stata Corporation, College Station, Texas, USA). Weighted mean difference (WMD) was used for analyzing the summary estimates, as the outcome measure was the same for each analysis. The statistical heterogeneity was assessed using the Q and I^2^ statistics (significance was set at p<0.10 or I^2^>50%). A random-effects model was used when significant heterogeneity was observed; otherwise, a fixed effects model was utilized. Furthermore, to explore the possible sources of heterogeneity in CPAP treatment effects, sensitive analysis and subgroup analysis were conducted. Publication bias was visually evaluated using funnel plots and statistically assessed using Egger’s and Begg’s tests and the ‘trim and fill’ method. A p value <0.05 was adopted as statistically significant.

## Results

### Searching results

**Fig 1:**
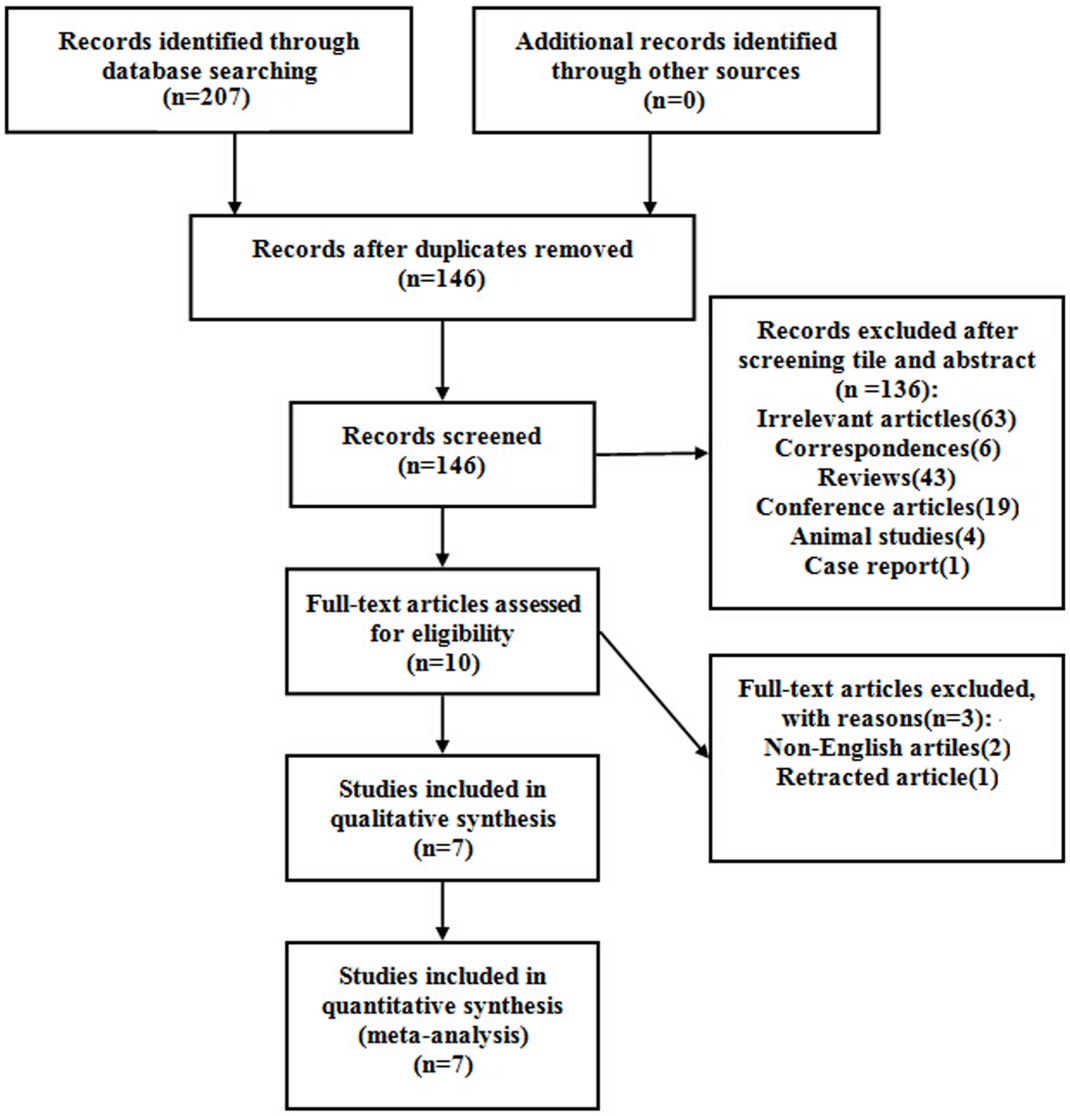
Flow diagram of study selection.

After excluding duplicates, our search identified 146 references. Of these, 10 studies were considered to be potentially relevant after a review of the titles and abstracts. Among the 10 studies, 3 studies were excluded from the sample for the following reasons: 2 were non-English studies [16, 17], 1 was retracted article [18]. Finally, 7 studies were included in the meta-analysis. The detailed steps of the literature search were presented in Fig 1.

### Characteristics of the studies

A total of 167 cases from the 7 identified studies were included in this meta-analysis. Two studies were randomized clinical trials (RCTs) [15, 19]; the remaining studies were observational [12, 14, 20–22]. One study [12] reported results separately for the 6 month group and the 12 month group, and only the data of the 12-month group was extracted. The characteristics of the 7 included studies and the patients’ characteristics were summarized in Tables 1 and 2, respectively. Two RCTs are summarized in Table 3.

**Table 1:** Characteristics of include studies.

Study	Year	Nation	Sample size/male	Inclusion criteria	Ventilation duration/night (h)	Therapy duration	Carotid IMT measurement	Study design
Drager	2007	Brazil	12/12	AHI>30	6±0.6	4M	Carotid IMT was measured at the thickest point, on the near and far walls with a specially designed computer program.	RCT
Li	2009	China	20/20	AHI≥15	6–8	3M	Carotid IMT was defined as the distance from the leading edge of the first echogenic line to the leading edge of the second echogenic line in the sonographic image.	Observational study
Hui	2012	Hong Kong	28/25	AHI≥5	4.7±2.1	12M	Carotid IMT was defined as the distance between the leading edge of the luminal echo to that of the media/adventitia echo and analyzed with a computerized edge-detection system.	Observational study
Agha	2014	Egypt	17/NR	AHI≥15	NR	6M	Carotid IMT was defined as the distance between the leading edge of the luminal echo to that of the media/adventitia echo.	Observational study
Kostopoulos	2016	Greece	25/NR	AHI≥15	>4	3M	NR	Observational study
Ng	2016	Hong Kong	45/32	AHI≥5	4.2±2.1	3M	Carotid IMT measurements were obtained at the thickest point (plaque-free section) on the far wall of distal common carotid artery, within 1.5 cm proximal to the flow divider.	RCT
Amin	2016	Kuwait	20/15	AHI≥5	NR	6M	Carotid IMT was defined as the distance between the leading edge of the luminal echo to that of the media/adventitia echo.	Observational study

Abbreviation: RCT = randomized controlled trial, AHI = apnea-hypopnea index, NR = not reported, h = hour, M = month, IMT = intima-media thickness.

**Table 2:** Patients’ characteristics of the trials included in the meta-analysis.

Study	Age	AHI	LowSO_2_	Pre-BMI	Post-BMI	Pre-CPAP carotid IMT(mm)	Post-CPAP carotid IMT(mm)
Drager	44 ±7	56 ±22	74 ± 11	29.9 ± 3.0	29.8±3.0	0.707 ±0.105	0.645± 0.095
Li	NR	54.25±22.78	71.78±13.98	NR	NR	0.90±0.19	0.88±0.16
Hui	48.8±9.5	39.0 ±19.0	74.7 ±11.1	28.2±3.7	28.3±4.2	0.758±0.159	0.705±0.106
Agha	NR	NR	NR	NR	NR	1.31±0.4	0.99±0.21
Kostopoulos	47.2±10.6(N = 28)	43.2(N = 28)	76.2(N = 28)	34.05 ±7.7(N = 28)	NR	0.696 ±0.168	0.7±0.163
Ng	50.3 ±10.1	30.6 ±21.4	79.6± 10.8	28.2± 3.9	NR	0.74 ±0.21	0.77±0.19
Amin	51±11	51 ±17	72±13	33.7±5.4	NR	0.702±0.134	0.587±0.134

Abbreviation: BMI = body mass Index, AHI = apnea-hypopnea index, LowSO_2_ = lowest O_2_ saturation, CPAP = continuous positive airway pressure, IMT = intima-media thickness, NR = not reported.

**Table 3:** Characteristics of two randomized controlled trials.

Author	Year	Study design	Treatment group		Control group			Jadad score
			Sample size	Change from baseline carotid IMT(mm)	type	Sample size	Change from baseline carotid IMT(mm)	
Drager	2007	Parallel	12	-0.062±0.1	No treatment	12	0.008±0.157	3
Ng	2016	Parallel	45	0.03±0.2	Sham CPAP	45	-0.03±0.1	5

Abbreviation: IMT = intima-media thickness, CPAP = continuous positive airway pressure.

### Pool analysis

**Fig 2:**
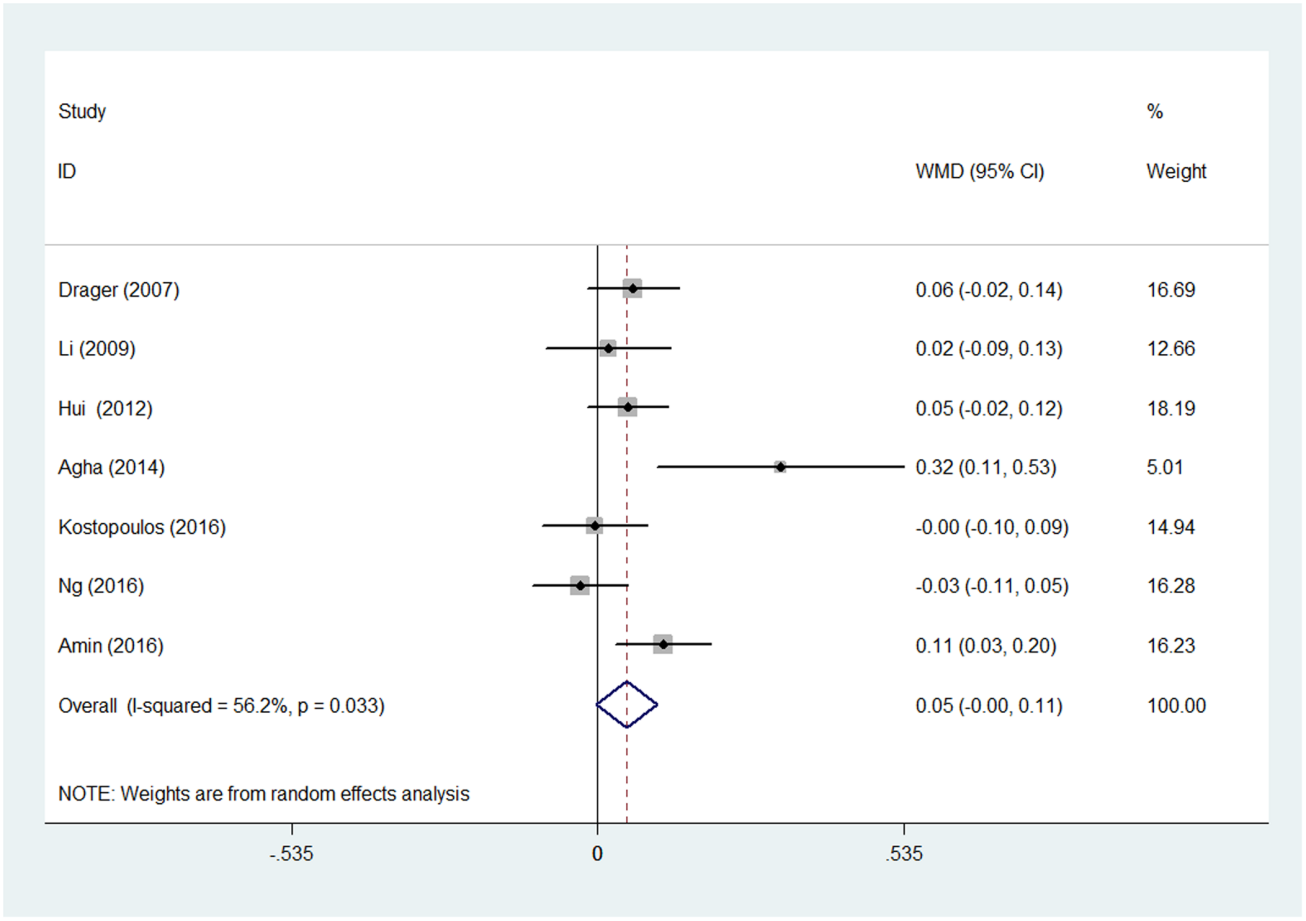
Forest plot for the change in carotid IMT before and after CPAP treatment. Abbreviation: CPAP = continuous positive airway pressure, IMT = intima-media thickness WMD = weighted mean difference.

**Fig 3:**
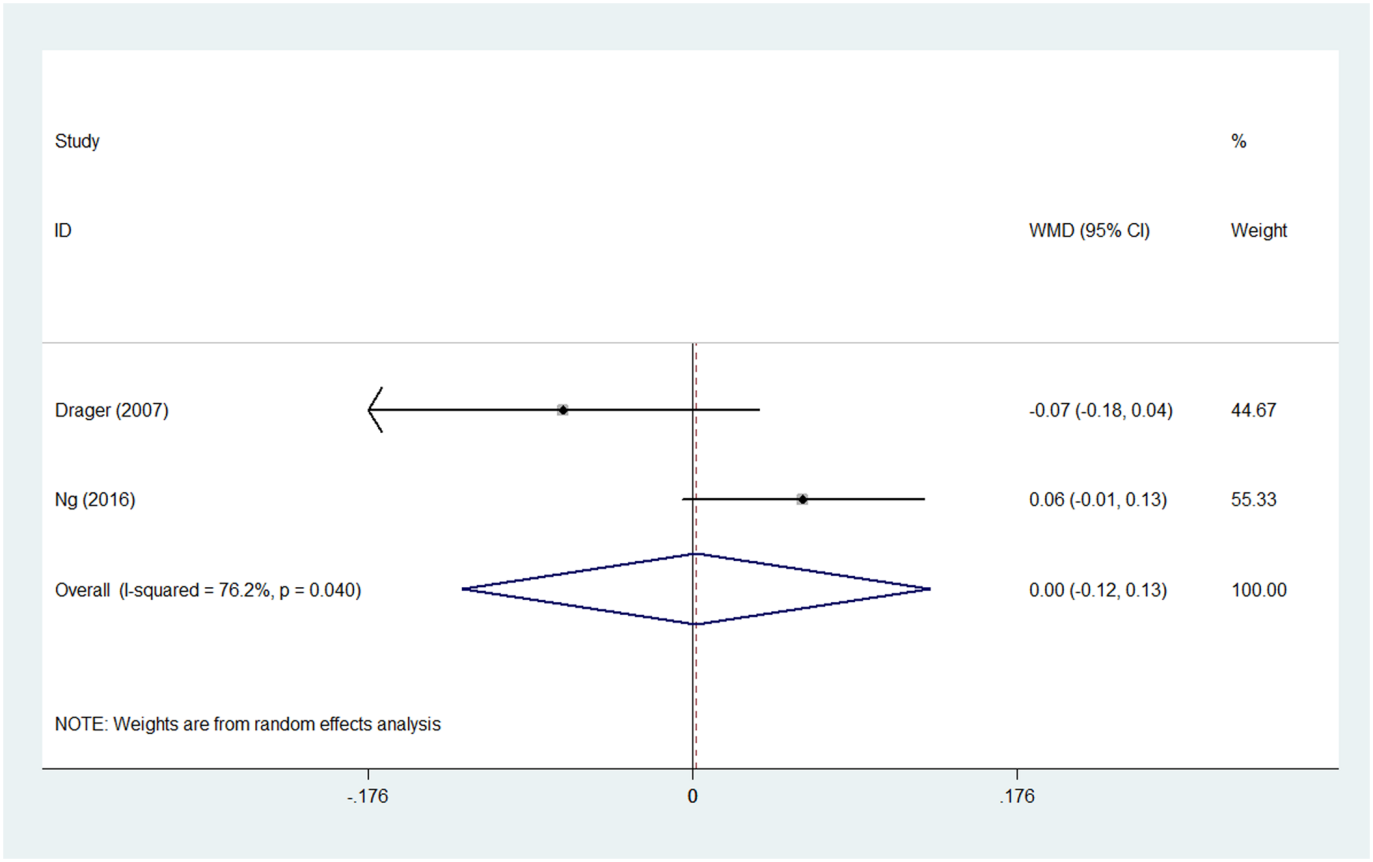
Forest plot for the change in carotid IMT between CPAP treatment group and control group in two RCTs. Abbreviation: CPAP = continuous positive airway pressure, IMT = intima-media thickness WMD = weighted mean difference.

Significant heterogeneity was detected among the included studies (chi-squared = 13.69, p = 0.033; I^2^ = 56.2%). Therefore, a random-effect model was used for the pooled analysis. No significant difference in carotid IMT in OSA patients was observed before and after CPAP treatment after pooling the data with meta-analysis (WMD = 0.052, 95% confidence interval (CI) = −0.002 to 0.105, z = 1.90, p = 0.057) (Fig 2). Meta-analysis of the two RCTs showed that carotid IMT was no changed in CPAP group when compared with control group. (WMD = 0.002 95% CI = −0.125 to 0.129, z = 0.03, p = 0.976, random effects model) (Fig 3).

### Sensitivity and subgroup analyses

**Fig 4:**
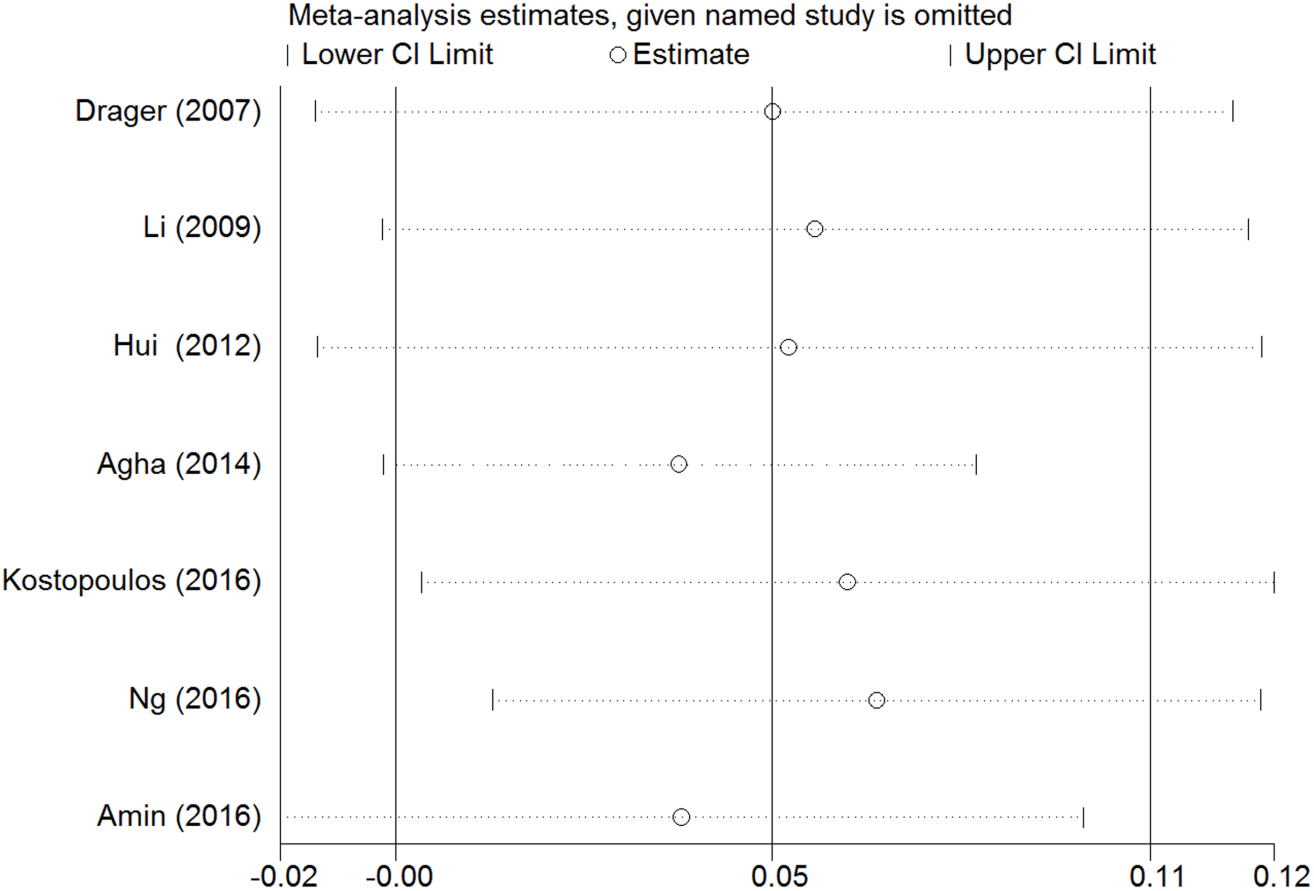
Sensitivity analysis of the included studies. Abbreviation: CI = confidence interval.

Sensitivity analysis showed that removal of each single study did not alter the overall results of pooled analyses (Fig 4). Subgroup analysis revealed that carotid IMT was significantly decreased after CPAP use in more severe OSA patients (AHI≥50) (WMD = 0.073, 95% CI = 0.022 to 0.124, z = 2.80, p = 0.005) and patients with therapeutic duration ≥6 months (WMD = 0.121, 95% CI = 0.019 to 0.223, z = 2.32, p = 0.021). While the differences in baseline BMI, age, and sample size did not affect CPAP efficacy. The detailed results of the subgroup analyses are presented in Table 4.

**Table 4:** The results of subgroup analyses.

Subgroup	Number of studies/patients	Heterogeneity			WMD			
		X^2^	P	I^2^(%)	WMD	95%CI	Z	P
Age								
<50	3/65	1.30	0.523	0.0	0.042	-0.004to0.088	1.78	0.075
≥50	2/65	5.88	0.015	83.0	0.042	-0.100to0.185	0.59	0.558
BMI								
<30	3/85	3.03	0.219	34.1	0.030	-0.025to0.086	1.08	0.279
≥30	2/45	3.55	0.059	71.8	0.057	-0.059to0.174	0.96	0.336
AHI								
<50	3/98	2.40	0.302	16.5	0.011	-0.040to0.062	0.43	0.667
≥50	3/52	1.97	0.374	0.0	0.073	0.022to0.124	2.80	0.005
Follow time (months)								
<6	4/102	2.62	0.454	0.0	0.013	-0.032to0.057	0.57	0.571
≥6	3/65	5.79	0.055	65.4	0.121	0.019to0.223	2.32	0.021
Sample size								
<20	2/29	4.87	0.027	79.5	0.171	-0.079to0.421	1.34	0.180
≥20	5/138	6.95	0.139	42.4	0.033	-0.018to0.084	1.28	0.202

Abbreviation: WMD = weighted mean difference, BMI = body mass index, AHI = apnea-hypopnea index.

### Publication bias

**Fig 5:**
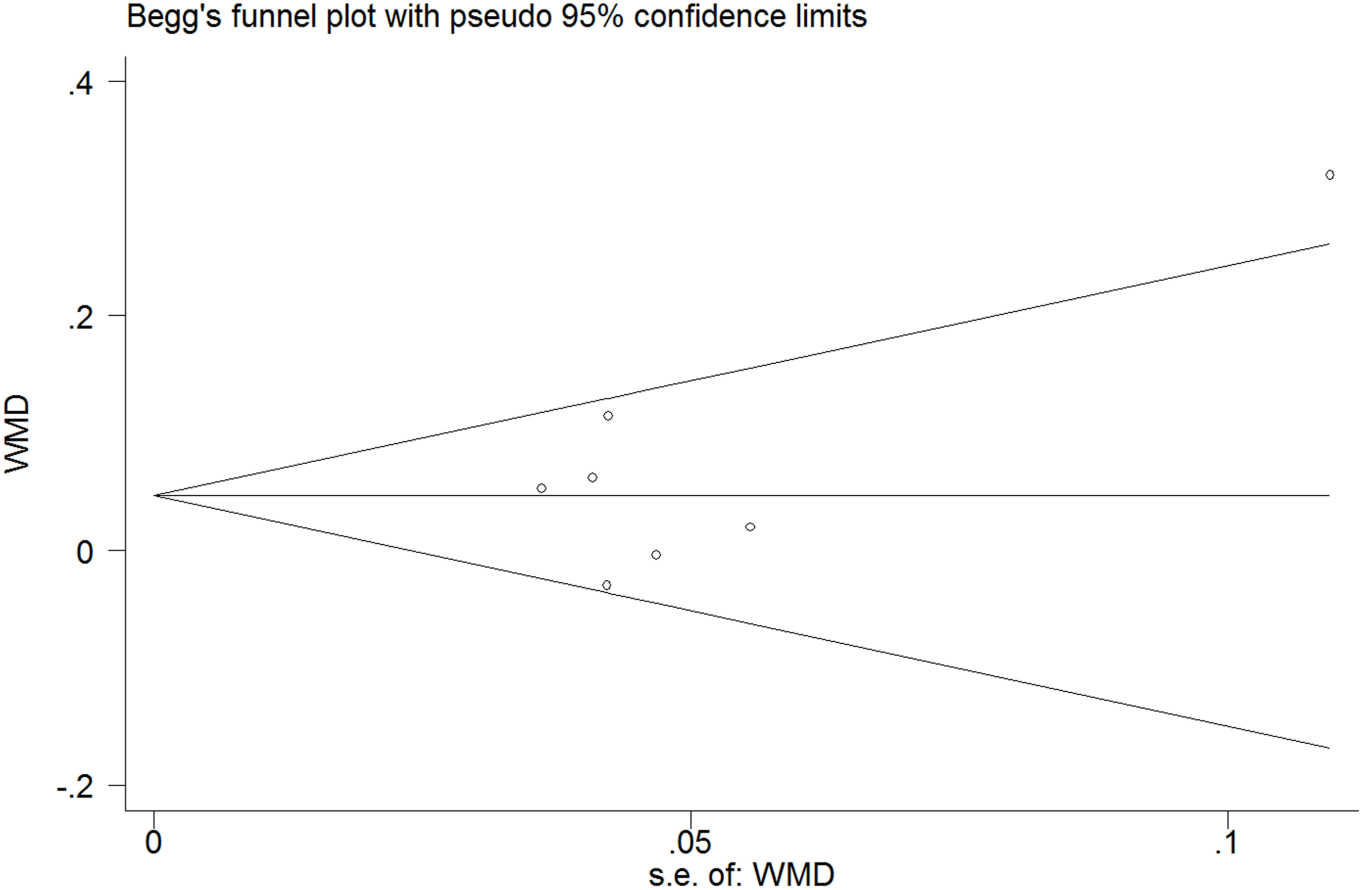
Funnel plots for assessing publication bias of studies included. SE = standard error, WMD = weighted mean difference.

The publication bias was not considered significant (Begg’s test, p = 0.548; Egger’s test, p = 0.245) (Fig 5). In addition, the trim-and-fill method showed that no study needed to be statistically corrected for funnel plot asymmetry.

## Discussion

To our knowledge, this is the first meta-analysis to determine the impact of OSA treatment with CPAP on carotid IMT. The findings of the present meta-analysis comprising of 167 patients suggested that CPAP had no impact on carotid IMT, an early marker of atherosclerosis, in OSA patients. The results were further confirmed by the pool analysis of two RCTs. However, subgroup analyses showed that CPAP was associated with a statistically significant decrease on carotid IMT in more severe OSA patients (AHI≥50) and patients with therapeutic duration ≥6 months.

Carotid IMT as assessed with carotid ultrasonography is a sensitive and reproducible marker for early and subclinical atherosclerosis. Carotid IMT greater than 0.8 mm is a marker of increased cardiovascular risk according to some studies [23–25]. Polak et al [5] followed 2965 members of the Framingham Offspring Study cohort for an average of 7.2 years and found that the maximum internal and mean common carotid-artery IMT both predicted cardiovascular outcomes. A meta-analysis including 1196 patients from 15 articles showed that a 0.1 mm increase in carotid IMT was predictive for myocardial infarction (HR 1.15, 95% CI 1.12–1.18) and for stroke (HR 1.17, 95% CI 1.15–1.21) [26]. It is also suggested that carotid stenosis may contribute to the development of cognitive dysfunction [7, 8]. In a study including 127 bilateral asymptomatic carotid stenosis, 150 unilateral asymptomatic carotid stenosis and 56 no carotid stenosis subjects, Balucani [8] found that subjects with bilateral asymptomatic carotid stenosis and subjects with unilateral asymptomatic carotid stenosis are more likely to have cognitive dysfunction compared to subjects with no carotid stenosis.

The relationship between OSA and carotid IMT has been well established in the previous literature [9–11]. In a study enrolling mild to moderate OSA, severe OSA and control groups matched for age, sex, and BMI, Drager et al [9] demonstrated a higher carotid IMT in severe OSA patients compared to mild to moderate OSA patients and non-OSA controls. Further multivariate analyses showed that the AHI correlated independently with IMT variability. A cross-sectional analysis of 83 patients identified that the severity of oxygen desaturation was one of the best predictors for carotid IMT and plaque occurrence in OSA patients [10]. In a study investigating the relationship between OSA and impairment of functional (Breath-Holding Index) and anatomic (IMT) characteristics of cerebral vessels, Buratti et al [27] found that pathological values of IMT and Breath-Holding Index were significantly associated with the presence of OSA after multivariate analysis (IMT > 1.0 mm: OR 2.98, 95%CI: 1.37–6.46; BHI < 0.69: OR 5.25, 95%CI: 2.35–11.74). A recent meta-analysis including 18 studies comparing carotid IMT of patients with OSA versus non-OSA patients found that patients with OSA had a significantly higher carotid IMT than healthy controls. After adjustment for several major confounders, OSA was an independent risk factor for carotid IMT [28]. OSA may promote carotid atherosclerosis through several mechanisms: intermittent hypoxemia, sympathetic nervous system overactivity, oxidative stress, endothelial dysfunction, lipid peroxidation and abnormal production of inflammatory cytokines [3, 29, 30].

CPAP treatment can effectively relieve obstruction of the airway, reversing OSA-associated hypoxia, sympathetic activation, oxidative stress, inflammatory cytokine levels [31, 32]. All of these factors may contribute to carotid atherosclerosis observed in patient with OSA. Hence, it was speculated to be beneficial to decrease carotid IMT in OSA patients. However, the present meta-analysis suggested that CPAP had no impact on carotid IMT in OSA patients. This conclusion was further supported by the pool analysis of the two RCTs. The results of subgroup analyses showed that carotid IMT was significantly decreased in more severe OSA patients and patients with longer therapy duration. Based on above findings, we believed that the therapy duration and baseline severity of OSA was the important factors influencing the effectiveness of CPAP treatment. A minimum of 6 months of CPAP was required to decrease carotid IMT in OSA patients. A significant decrease of carotid IMT was observed in patients with more severe OSA. This could be because these patients have more substantial physiologic abnormalities with greater degrees of nocturnal hypoxemia and higher baseline carotid IMT. Another reason to explain the result could be that compliance with CPAP treatment may be better in severe OSA patients compared with less severe OSA.

The present analysis had several limitations that warrant additional comment. First, it should be noted that although the total sample size in this meta-analysis was relatively small. Second, most of the studies included in this meta-analysis were observational rather than RCTs. Third, the follow up was relatively short in the majority of these studies, with only one study reached up to 12 months. Fourth, there was some evidence of heterogeneity among the studies, but no exact source of heterogeneity was identified. Furthermore, only papers published in English were enrolled, it may cause potential publication bias. Finally, since periodic limb movements in sleep is a risk factor for cerebral small vessel disease and is frequently associated with OSA [33, 34], periodic limb movements may be a founding factor. However, all of the studies included did not exclude the patients with periodic limb movements.

In summary, this meta-analysis did not support for a favorable effect of CPAP treatment on decreasing carotid IMT among patients with OSA. However, carotid IMT was significantly decreased after CPAP treatment in more severe OSA patients and patients with longer CPAP usage. Further Longer-term randomized trials are needed to fully characterize the efficacy of CPAP therapy on carotid IMT in OSA.

## Supporting information

S1 File PRISMA checklist. (DOC)
